# A Novel Hybrid Meta-Heuristic Algorithm Based on the Cross-Entropy Method and Firefly Algorithm for Global Optimization

**DOI:** 10.3390/e21050494

**Published:** 2019-05-14

**Authors:** Guocheng Li, Pei Liu, Chengyi Le, Benda Zhou

**Affiliations:** 1 School of Finance and Mathematics, West Anhui University, Lu’an 237012, China; 2 Institute of Financial Risk Intelligent Control and Prevention, West Anhui University, Lu’an 237012, China; 3 College of Computer Science, Sichuan University, Chengdu 610065, China; 4 School of Economic & Management, East China Jiaotong University, Nanchang 330013, China

**Keywords:** global optimization, meta-heuristic, firefly algorithm, cross-entropy method, co-evolution

## Abstract

Global optimization, especially on a large scale, is challenging to solve due to its nonlinearity and multimodality. In this paper, in order to enhance the global searching ability of the firefly algorithm (FA) inspired by bionics, a novel hybrid meta-heuristic algorithm is proposed by embedding the cross-entropy (CE) method into the firefly algorithm. With adaptive smoothing and co-evolution, the proposed method fully absorbs the ergodicity, adaptability and robustness of the cross-entropy method. The new hybrid algorithm achieves an effective balance between exploration and exploitation to avoid falling into a local optimum, enhance its global searching ability, and improve its convergence rate. The results of numeral experiments show that the new hybrid algorithm possesses more powerful global search capacity, higher optimization precision, and stronger robustness.

## 1. Introduction

In many tasks or applications, global optimization plays a vital role, such as in power systems, industrial design, image processing, biological engineering, job-shop scheduling, economic dispatch and financial markets. In this paper, we focus our attention on unconstrained optimization problems which can be formulated as min$f\left(x\right):x\in R^{n}$, where $f:R^{n}\mapsto R$ and *n* refers to the problems’ dimension [1]. Traditional optimization methods such as the gradient-based methods usually struggle to deal with these challenging problems due to the objective function f(x) can be nonlinearity, multimodality and non-convexity [2,3]. Thus, for decades, researchers have explored many derivative-free optimization methods to solve them. Generally, these optimization methods can be divided into two main classes: deterministic algorithms and stochastic algorithms [3,4]. The former, such as the Hill-Climbing [5], Newton–Raphson [6], DIRECT Algorithm [7], and Geometric and Information Global Optimization Methods with local tuning or local improvement [8,9], can get the same final results if the same set of initial values are used at the beginning [10]. However, the latter such as two well-known algorithms—Genetic Algorithm (GA) [11] and Particle Swarm Optimization (PSO) [12]—often use some randomness in their strategies which can enable the algorithm to escape from the local optima to search more regions on a global scale [10], and which have become very popular for solving real-life problems [3].

In the past two decades, meta-heuristics based on evolutionary computation and swarm intelligence have emerged and become prevalent, such as Ant Colony Optimization (ACO) [13], Differential Evolution (DE) [14], Harmony Search (HS) [15], Bacterial Foraging Optimization Algorithm (BFOA) [16], Honey Bees Mating Optimization (HBMO) [17], Artificial Bee Colony (ABC) [18], Biogeography-Based Optimization (BBO) [19], Gravitational Search Algorithm (GSA) [20], Firefly Algorithm (FA) [21], Cuckoo Search (CS) [22], Bat Algorithm (BA) [23], Grey Wolf Optimizer (GWO) [24], Ant Lion Optimizer (ALO) [25], Moth Flame Optimizer (MFO) [26], Dragonfly Algorithm (DA) [27], Whale Optimization Algorithm (WOA) [28], Salp Swarm Algorithm (SSA) [29], Crow Search Algorithm (CSA) [30], Polar Bear Optimization (PBO) [31], Tree Growth Algorithm (TGA) [32], and Butterfly Optimization Algorithm (BOA) [33]. Meta-heuristic algorithms have been widely adopted to deal with global optimization and engineering optimization problems, and have attracted much attention as effective tools for optimization.

However, superior performance for any meta-heuristic algorithm is a target. They perform well when dealing with certain optimization problems but are not ideal in most cases [34]. In order to overcome this shortcoming, many hybrid meta-heuristic algorithms trying to combine meta-heuristics and exact algorithms or other meta-heuristics have been proposed to solve more complicated optimization problems, such as Hybrid Genetic Algorithm with Particle Swarm Optimization [35], Hybrid Particle Swarm and Ant Colony Optimization [36], Hybrid Particle Swarm Optimization with Gravitational Search Algorithm [37], Hybrid Evolutionary Firefly Algorithm [38], Hybrid Artificial Bee Colony with Firefly Algorithm [39], Hybrid Firefly-Genetic Algorithm [40], Hybrid Firefly Algorithm with Differential Evolution [10], Simulated Annealing Gaussian Bat Algorithm [41], Hybrid Harmony Search with Cuckoo Search [42], Hybrid Harmony Search with Artificial Bee Colony Algorithm [43], and Hybrid Whale Optimization Algorithm with Simulated Annealing [44]. These hybrid meta-heuristic algorithms have been successfully applied in function optimization, engineering optimization, portfolio selection, shop scheduling optimization, and feature selection.

Based on co-evolution, this paper explores a new hybrid meta-heuristic algorithm combining the cross-entropy (CE) method and the firefly algorithm (FA). The cross-entropy method was proposed by Rubinstein [45] in 1997 to solve rare event probability estimation in complex random networks, while the firefly algorithm (FA) was developed by Yang [21] and inspired by the flashing pattern of tropical fireflies in nature for multimodal optimization. The motivation of our new proposed hybrid algorithm is to improve the global search ability by embedding the cross-entropy method into the firefly algorithm to obtain an effective balance between exploration and exploitation.

The rest of the paper is organized as follows. In Section 2, CE and FA are briefly introduced, and their hybridization study is presented in Section 3. Numeral experiments and results are given in Section 4. Further analysis and a discussion of the performance of the new method are conducted in Section 5. In Section 6, the conclusions of the paper are presented.

## 2. Preliminaries

### 2.1. The Cross-Entropy Method

The cross-entropy (CE) method was proposed by Rubinstein [45] in 1997 based on Monte Carlo technology and uses Kullback–Leibler divergence to measure the cross-entropy between two sampling distributions, solve an optimization problem by minimizing them, and obtain the optimal probability distribution parameters. CE has excellent global optimization capability, good adaptability, and strong robustness. Thus, Yang regards it as a meta-heuristic algorithm [4]. However, due to the large sample size, it has the disadvantages of large computational cost and slow convergence rate. CE not only solves rare event probability estimation problems. It can also be used to solve complex optimization problems such as combination optimization [46,47,48], function optimization [46,48,49], engineering design [50], vehicle routing problems [51], and problems from other fields [52,53,54].

Let us consider the optimization problem as follows:

(1) $$\min S\left(x\right):X\in R^{n}\to R,$$

where *S* is a real-valued performance function on *X*.

Now, we associate the above problem with a probability distribution estimation problem, and the auxiliary problem is obtained:

(2) $$l\left(\gamma \right)=P_{\mu }\left(S\left(X\right)\leq \gamma \right)=E_{\mu }\left[I_{S(X)\leq \gamma }\right],$$

where $E_{\mu }$ is the expectation operator, γ is a threshold or level parameter, and *I* is the indicator function, whose value is 1 if S(X)≤γ and 0, otherwise. In order to reduce the number of samples, the importance sampling method is introduced in CE. Consequently, we can rewrite Equation (2) as

(3) $$l\left(\gamma \right)=\frac{1}{N}\sum_{i=1}^{N}I_{S(X)\leq \gamma }\frac{f(x^{i};v)}{g(x^{i})},$$

where $x^{i}$ is a random sample from f(x;v) with importance sampling density g(x). In order to obtain the optimal importance sampling density, the Kullback–Leibler divergence is employed to measure the distance between two densities, i.e., the cross-entropy, and the Kullback–Leibler divergence is minimized to obtain the optimal density $g^{*}\left(x\right)$, which is equivalent to solving the minimization problem [45]

(4) $$\min_{v}\frac{1}{N}\sum_{i=1}^{N}I_{S(X)\leq \gamma }\ln f\left(x^{i};v\right).$$

The main CE algorithm for optimization problems is summarized in Algorithm 1. 

**Algorithm 1:** CE for Optimization Problems**Begin**    Set t=0. Initialize the value of the probability distribution parameter ${\hat{v}}_{k}$.
   **while** (t<MaxGeneration)
        Generate $Y_{1},Y_{2},\ldots ,Y_{L}{∼}_{iid}f\left(x;{\hat{v}}_{k}\right)$. Evaluate and rank the sample. 
        Use the sample $Y_{1},Y_{2},\ldots ,Y_{L}$ to solve the problem given in Equation (4). Denote the solution by $\tilde{v}$. 
        Adaptive smoothing ${\hat{v}}_{k}$ is demoted by $\tilde{v}$. (5)$${\hat{v}}_{k+1}=\alpha \tilde{v}+\left(1-\alpha \right){\hat{v}}_{k},$$ where 0≤α≤1 is a smoothing parameter. 
        Set t=t+1. 
   **end while**
   Output the best solution. **End**

### 2.2. Firefly Algorithm

The firefly algorithm (FA) was proposed by Yang [21] and inspired by the unique light signal system of fireflies in nature. Fireflies use radiance as a signal to locate and attract the opposite sex, even to forage. Based on idealizing the flashing characteristics of fireflies, the firefly algorithm was formulated for solving optimization problems. Using this algorithm, random search and optimization can be performed within a certain range, such as the solution space. Through the movement of fireflies and the constant renewal of brightness and attraction, they are constantly approaching the best position and ultimately get the best solution to the problem. FA has attracted much attention and has been applied to many applications such as global optimization [55], multimodal optimization [21], multi-objective optimization [56], engineering design problems [57], scheduling problems [58], and other fields [59,60,61,62].

In order to design FA properly, two important issues need to be defined: the variation of light intensity and formulation of the attractiveness [21]. The light intensity of a firefly can be approximated as follows:

(6) $$I=I_{0}\times e^{-\gamma r_{ij}^{2}},$$

where $I_{0}$ represents the original light intensity and γ is a fixed light absorption coefficient. $r_{ij}$ indicates the distance between firefly *i* and firefly *j* and is defined as follows:

(7) $$r_{ij}=∥x_{i}-x_{j}∥=\sqrt{\sum_{k=1}^{d}{\left(x_{ik}-x_{jk}\right)}^{2}}.$$

The attractiveness of a firefly can be formulated as follows:

(8) $$\beta =\beta_{0}\times e^{-\gamma r_{ij}^{2}},$$

where $\beta_{0}$ represents the attractiveness at r=0, which is the maximum attractiveness. Due to the attractiveness from firefly *j*, the position of firefly *i* is updated as follows:

(9) $$s_{i}=s_{i}+\beta \times \left(s_{j}-s_{i}\right)+\lambda \times \left(rand-0.5\right),$$

where $s_{i}$ and $s_{j}$ are the positions of fireflies *i* and *j*, respectively. The step factor λ is a constant and satisfies 0<λ<1, and rand is a random number generator uniformly distributed in [0,1], which was later replaced by Lévy flight [55].

Based on the above, the main FA can be summarized in pseudo-code as Algorithm 2. 

**Algorithm 2:** Firefly Algorithm**Begin**    Objective function $f\left(x\right),x={\left(x_{1},x_{2},\ldots ,x_{d}\right)}^{T}$. 
   Initialize a population of fireflies $pop_{i}\left(i=1,2,\ldots ,n\right)$. 
   Calculate the fitness value $f(pop_{i})$ to determine the light intensity $I_{i}$ at $pop_{i}$. 
   Define light absorption coefficient γ. 
   **while** (t<MaxGeneration) 
        **for**
i=1:n all *n* fireflies 
             **for**
j=1:n all *n* fireflies 
                  **if** ($I_{j}>I_{i}$) 
                       Move firefly *i* towards *j* in all *d*-dimensions via Lévy flight. 
                  **end if**
                  Attractiveness varies with distance *r* via $-e^{-\gamma r^{2}}$. 
                  Evaluate new solutions and update light intensity.               **end for**
*j*
        **end for**
*i*
        Rank the fireflies and find the current best.    **end while**
   Output the best solution.
**End**

## 3. Novel Hybrid Cross-Entropy Method and Firefly Algorithm

In this section, the details of the new hybrid algorithm are presented. A meta-heuristic algorithm should have two main exploration and exploitation functions, and an excellent meta-heuristic algorithm should try to effectively balance them and achieve better performance [63]. The cross-entropy method based on the Monte Carlo technique has the advantages of strong global optimization ability, good adaptability, and robustness [46]. It also has obvious disadvantages of large sample size, high computational cost, and slow convergence. At the same time, the firefly algorithm based on bionics has the advantages of strong local search ability and fast convergence, but it tends to fall into a local optimum rather than obtaining a global optimal solution [21]. Based on a co-evolutionary technique, this paper explores constructing a new hybrid meta-heuristic algorithm, named the Cross-Entropy Firefly Algorithm (CEFA), by embedding the cross-entropy method into the firefly algorithm. The new method contains two optimization operators—the CE operator and FA operator—which implement information sharing between the CE sample and the FA population through co-evolution in each iteration. While the FA operator updates its population using the elite sample from CE to improve the population diversity, the CE operator uses the FA population to calculate the initial probability distribution parameters in order to speed up its convergence.

The new hybrid meta-heuristic algorithm based on a co-evolutionary technique preserves the advantage of fast convergence of the swarm intelligent bionic algorithm in local search. At the same time, it also makes full use of the global optimization ability of the cross-entropy stochastic optimization method. The introduction of a co-evolutionary technique not only makes the meta-heuristic algorithms from different backgrounds complement each other but also enhances their respective advantages. Therefore, it has strong global exploration capability and local exploitation capability, and can quickly converge to global optimal solution, which provides powerful algorithm support for complex function optimization or engineering optimization problems.

The pseudo-code of CEFA is described in Algorithm 3.

**Figure 1:**
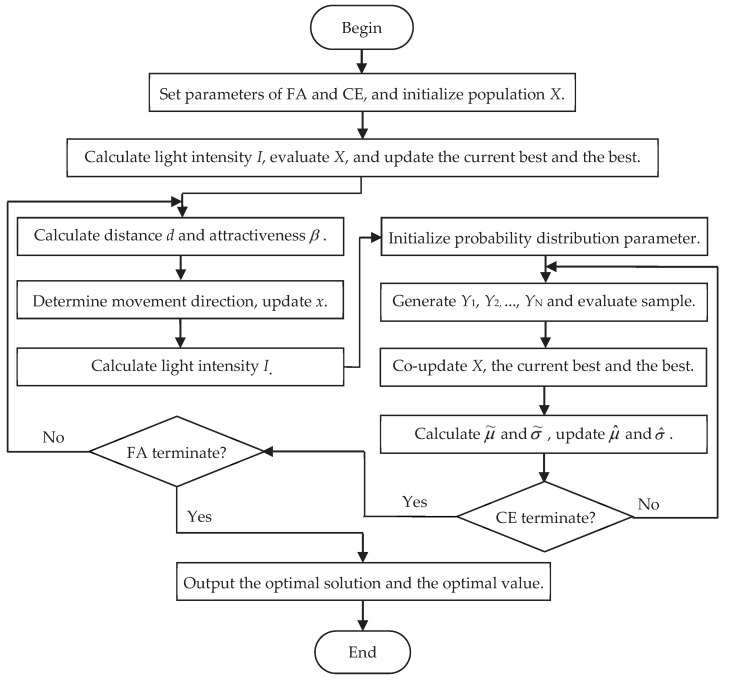
The flow chart of the Cross-Entropy Firefly Algorithm (CEFA).

In order to more clearly show the co-evolutionary process between the FA operator and the CE operator, the flow chart of CEFA is presented in Figure 1.

**Algorithm 3:** Cross-Entropy Firefly Algorithm**Begin**    Objective function $f\left(x\right),x={\left(x_{1},x_{2},\ldots ,x_{d}\right)}^{T}$. 
   Initialize a population of fireflies $X_{i}\left(i=1,2,\ldots ,n\right)$. 
   Calculate the fitness value $f(X_{i})$ to determine the light intensity $I_{i}$ at $X_{i}$. 
   Define light absorption coefficient γ. 
   **while** (t<MaxGeneration_FA) 
        **for**
i=1:n all *n* fireflies 
             **for**
j=1:n all *n* fireflies 
                  **if** ($I_{j}>I_{i}$) 
                       Move firefly *i* towards *j* in all *d*-dimensions via Lévy flight. 
                  **end if**
                  Attractiveness varies with distance *r* via $-e^{-\gamma r^{2}}$. 
                  Evaluate new solutions and update light intensity.              **end for**
*j*
        **end for**
*i*
        Rank the fireflies and find the current best.         Initialize the probability distribution parameter ${\hat{v}}_{k}$ by the population X. 
        **for**
k=1:MaxGeneration_CE
             Generate $Y_{1},Y_{2},\ldots ,Y_{N}{∼}_{iid}f\left(x;{\hat{v}}_{k}\right)$. Evaluate the sample Y. 
             Rank the population X and the sample Y together, update the current best. 
             Update the population X of FA and the elite sample $Y_{e}$ of CE. 
             Calculate the probability distribution parameter $\tilde{v}$ by the elite sample $Y_{e}$. 
             Update the probability distribution parameter via Equation (5).         **end for**
*k*
   **end while**
   Output the best solution.
**End**

## 4. Experiment and Results

### 4.1. Benchmark Functions

In this section, 23 standard testing functions utilized by many researchers [20,24,25,27,28,29] were employed to evaluate the performance of the proposed hybrid algorithm CEFA for numerical optimization problems. The benchmark functions including seven unimodal functions, six multimodal functions and ten fixed-dimension multimodal functions are described in Appendix A (Table A1). The unimodal functions were used to evaluate the exploitation and convergence of an algorithm, while the multimodal functions were used to benchmark the performance of exploration and local optima avoidance [25,27]. Further information on all the benchmark functions can be found in Yao et al. (1999) [64].

### 4.2. Experiment Setting

Three test experiments were performed using the proposed CEFA method, and the obtained numerical solutions were compared with those from FA [21], CE [45], GA [11], PSO [12], SSA [29], BOA [31], and Hybrid Firefly Algorithm (HFA) [10] on the benchmark functions. Further information on the experiments is shown in Table 1. For these experiments, the variants were coded in MATLAB R2018b, running on a PC with an Intel Core i7-8700 machine (Gainesville, FL, USA), 3.19 GHz CPU, and 16 GB of RAM.

Test experimental conditions and settings: (1) The population size of the FA operator in CEFA was set to 60 for Test 1 and 100 for Tests 2 and 3, while the sample size of the CE operator was 98. The maximum number of iterations of the FA operator in CEFA was 50, while the CE operator’s was 30 for Test 1 and 50 for Tests 2 and 3. (2) The population sizes of other algorithms for comparison were 100, and the maximum number of iterations were 1500 for Test 1 and 2500 for Tests 2 and 3. (3) All the other parameters of each algorithm were set to be as the same as the original reference. This experimental setup ensures fairness in comparison because the numbers of functional evaluations (NFEs) for all algorithms were the same in the same test.

It is well known that all the intelligent methods are based on a certain stochastic distribution, so 30 independent runs were carried out for each method on each test function in order to statistically evaluate the proposed hybrid algorithm. The average value and standard deviation of the best approximated solution in the last iteration are introduced to compare the overall performance of the algorithms.

### 4.3. Results and Comparisons

The results of Test 1 are shown in Table 2. The winner (best value) is identified in bold. Among the results, the average value was used to evaluate the overall quality of the solution, reflecting the average solution accuracy of the algorithm, and the standard deviation was used to evaluate the stability of the algorithm. From Table 2, we can see the following: (1) The proposed algorithm outperforms FA, CE, GA, PSO, and SSA on almost all seven unimodal functions and six multimodal functions, while it is superior to BOA and HFA for the majority of them. This indicates that CEFA has good performance in terms of exploitation, exploration and local optima avoidance. (2) CEFA provides very competitive results in most of the ten fixed-dimension multimodal functions and tends to outperform other algorithms. The advantages of CEFA have not been fully demonstrated when solving low-dimensional function optimization problems.

**Figure 2:**
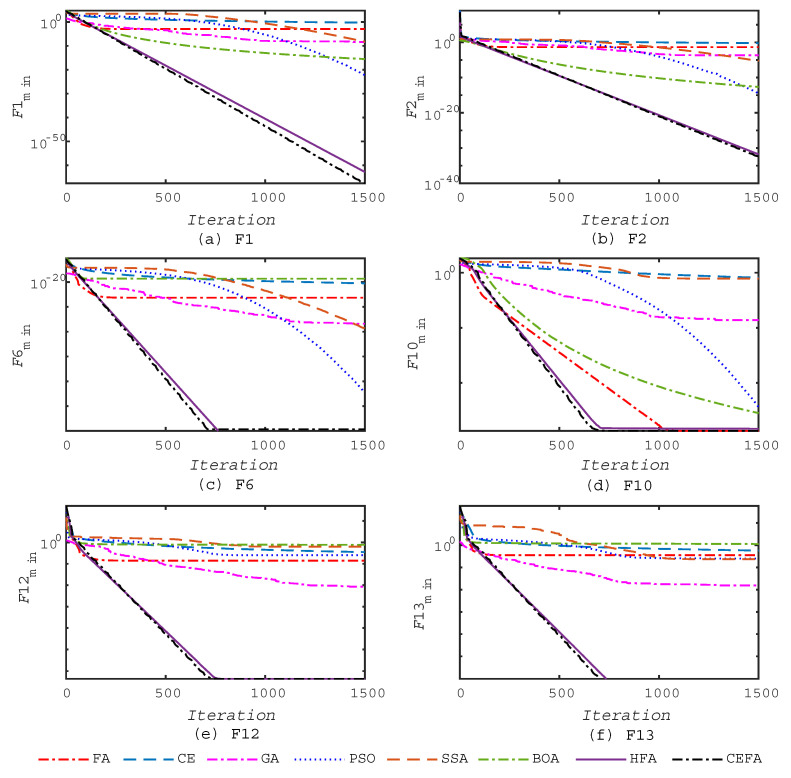
Convergence of algorithms on some of the benchmark functions in Test 1.

The progress of the average best value over 30 runs for the benchmark functions F1, F2, F6, F10, F12, and F13 is shown in Figure 2; it shows that the proposed CEFA tends to find the global optimum significantly faster than other algorithms and has a higher convergence rate. This is due to the employed co-evolutionary mechanisms adopted between CE and FA to place emphasis on the local search and exploitation as the iteration number increases, which highly accelerate the convergence towards the optimum in the final steps of the iterations.

Tests 2 and 3 were intended to further explore the advantages of the CEFA algorithm in solving large-scale optimization problems. The test results are shown in Table 3 and Table 4. Both of them show that the proposed algorithm outperforms GA, PSO, and SSA on all test problems, except for one problem with a slight difference from GA or PSO and provides very competitive results compared to BOA and HFA on the majority of multimodal functions. The superior performance of the proposed method in solving large-scale optimization problems is attributed to a good balance between exploration and exploitation, which also enhances CEFA’s exploration and exploitation capabilities to focus on the high-performance areas of the search space.

**Figure 3:**
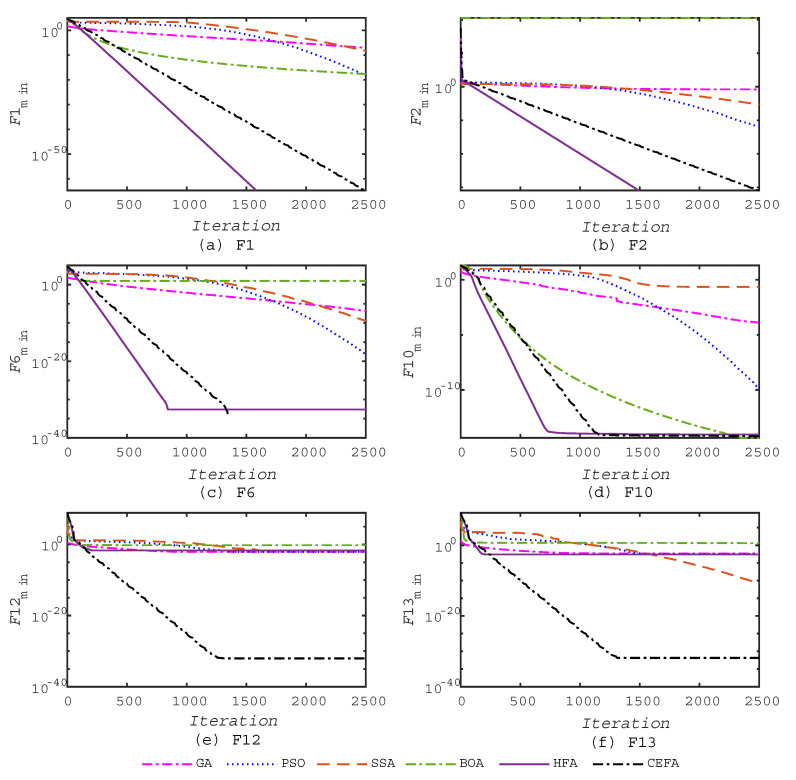
Convergence of algorithms on some of the benchmark functions in Test 2.

**Figure 4:**
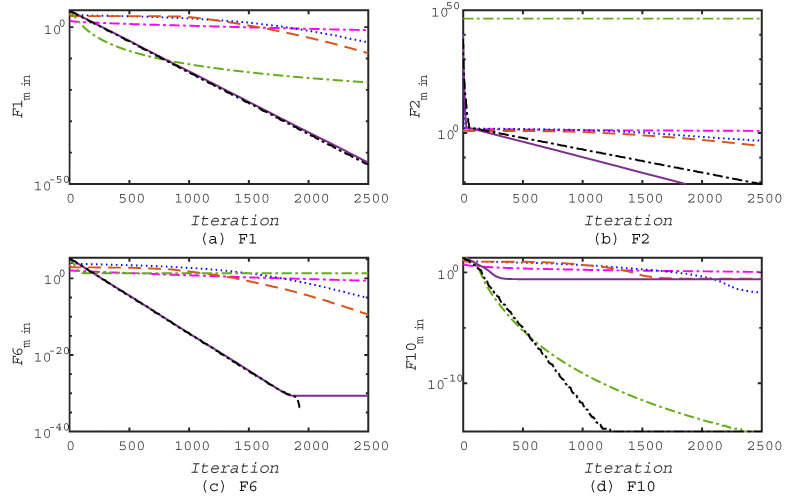

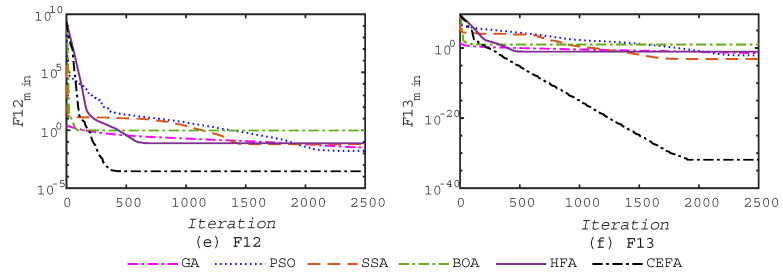
Convergence of algorithms on some of the benchmark functions in Test 3.

In addition, the good convergence speed of the proposed CEFA algorithm could be concluded from Figure 3 and Figure 4 when solving large-scale optimization problems, in which the same six functions, F1, F2, F6, F10, F12, and F13, were selected from the benchmark functions for comparison. From these, we can see that the local optima avoidance of this algorithm is satisfactory since it is able to avoid all of the local optima and approximate the global optima on the majority of the multimodal test functions. These results reaffirm that the operators of CEFA appropriately balance exploration and exploitation to handle difficulty in a challenging and high-dimensional search space.

## 5. Discussion

### 5.1. Advantage Analysis of CEFA

The main reasons for the superior performance of the proposed hybrid meta-heuristic algorithm based on CE and FA in solving complex numerical optimization problems may be summarized as follows:

- CE is a global stochastic optimization method based on Monte Carlo technology, and has the advantages of randomness, adaptability, and robustness; this makes the FA population in the hybrid algorithm have good diversity so that it can effectively overcome its tendency to fall into a local optimum and improve its global optimization ability.
- FA mimicking the flashing mechanism of fireflies in nature has the advantage of fast convergence. With co-evolution, CEFA uses the superior individuals obtained by the FA operator to update the probability distribution parameters in the CE operator during the iterative process, which improves the convergence rate of the CE operator.
- The hybrid meta-heuristic algorithm CEFA introduces the co-evolutionary technique to collaboratively update the FA population and the probability distribution parameters in CE, which obtains a good balance between exploration and exploitation, and has excellent performance in terms of exploitation, exploration, and local optima avoidance in solving complex numerical optimization problems. In addition, the proposed CEFA can effectively solve complex high-dimensional optimization problems due to the superior performance of CE in solving them.

### 5.2. Efficiency Analysis of Co-Evolution

The proposed hybrid meta-heuristic algorithm CEFA employs co-evolutionary technology to achieve a good balance between exploration and exploitation. The application of this co-evolutionary technology can be summarized by three aspects: (1) The CE operator and the FA operator collaboratively update the optimal solution and optimal value. (2) The initial probability distribution parameters of the CE operator during the iterative process are updated with the population of the FA operator. (3) The result of each iteration of the CE operator updates the current population of the FA operator to obtain the best population.

**Figure 5:**
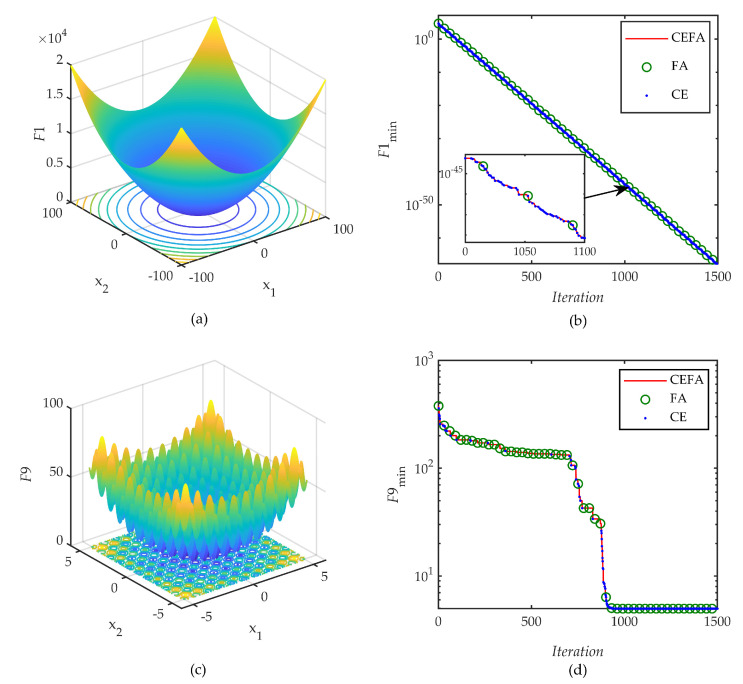
Efficiency analysis of co-evolution: (**a**,**c**) two-dimensional versions of F1 and F9; (**b**,**d**) FA and CE co-update the current best in CEFA’s iterative process.

Figure 5 shows the specific process of co-evolution when the hybrid algorithm is used to solve F1 and F9 selected from the benchmark functions, where “o” is the optimal function value updated by the FA operator and “.” is updated by the CE operator. This fully demonstrates that the co-evolutionary technology can be well implemented in the proposed method and the optimal function value is collaboratively updated by the two operators FA and CE during the iterative process.

### 5.3. Parameter Analysis of CEFA

In the proposed hybrid meta-heuristic algorithm, the numbers of iterations of the operators CE and FA are two key parameters that affect its performance in solving numerical optimization problems. To this end, this paper took F1 (dimension *d* = 30) as an example, and used the experimental method to explore the influence of their different combinations on the optimization results. The specific experiment was set as follows: the number of iterations $N_{1}$ of the CE operator was set to 1, 5, 10, 30, 50, 100, 200, or 300, while the number of iterations of the FA operator $N_{2}$ took values of 30, 50, 100, 200, 500, or 1000, and all the other parameters were the same as before. The results were averaged over 30 runs and the average optimal function value and time consumption are reported in Table 5.

Table 5 shows that the hybrid algorithm can adjust the number of iterations $N_{1}$ and $N_{2}$ of the two operators in solving the specific optimization problem to achieve higher accuracy. The values of $N_{1}$ and $N_{2}$ are determined by the characteristics and complexity of the given optimization problem, and they are generally between 30 and 100.

### 5.4. Performance of CEFA for High-Dimensional Function Optimization Problems

In order to further explore the influence of search space dimension on the optimization performance and convergence rate of CEFA when solving high-dimensional function optimization problems, this paper selected the standard GA, PSO, SSA, BOA, and HFA as comparison objects to test F1 from the benchmark functions. The dimension of the search space was increased from 10 to 200 in steps of 10.

**Figure 6:**
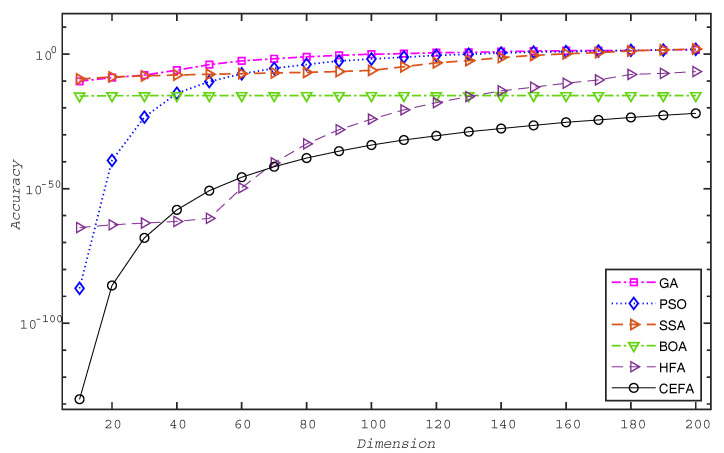
Comparison of optimization accuracy of different search space dimensions.

It can be seen from Figure 6 that the accuracy of the proposed CEFA is not greatly affected by the increase of the dimension of the search space, which is obviously different from GA, PSO, and SSA. It can be also seen that BOA has the same advantage, but its solution accuracy is not as high as that of CEFA. As the dimensions of the search space increase, for example, it is greater than 70 for F1, CEFA obtains more accurate results than HFA. This may provide a new and effective way for solving high-dimensional function optimization problems.

## 6. Conclusions

Global optimization problems are challenging to solve due to their nonlinearity and multimodality. In this paper, based on the firefly algorithm and the cross-entropy method, a novel hybrid meta-heuristic algorithm was constructed. In order to enhance the global search ability of the proposed method, the co-evolutionary technique was introduced to obtain an efficient balance between exploration and exploitation. The benchmark functions are employed to evaluate the performance of the proposed hybrid algorithm CEFA for numerical optimization problems. The results of the numeral experiments show that the new method provides very competitive results and possesses more powerful global search capacity, higher optimization precision, and stronger robustness. Furthermore, the new method exhibits excellent performance in solving high-dimensional function optimization problems. In addition, for future research, a discrete version of CEFA will be developed to solve combinatorial optimization problems. 

## Figures and Tables

**Table 1:** Information about the three test experiments.

Name	Functions	Dimension	Comparisons
Test 1	F1–F23	2–30	FA, CE, GA, PSO, SSA, BOA, HFA, CEFA
Test 2	F1–F13	50	GA, PSO, SSA, BOA, HFA, CEFA
Test 3	F1–F13	100	GA, PSO, SSA, BOA, HFA, CEFA

**Table 2:** Comparison of the optimization results obtained in Test 1 (d= 2–30).

Fun.	Meas.	FA	CE	GA	PSO	SSA	BOA	HFA	CEFA
F1	Aver.	$1.23\times 10^{-03}$	$5.45\times 10^{-01}$	$1.10\times 10^{-09}$	$3.18\times 10^{-23}$	$5.92\times 10^{-09}$	$3.09\times 10^{-16}$	$1.64\times 10^{-63}$	$3.04\times 10^{-68}$
	Stdev.	$4.35\times 10^{-03}$	$6.72\times 10^{-02}$	$3.48\times 10^{-09}$	$8.40\times 10^{-23}$	$8.80\times 10^{-10}$	$1.40\times 10^{-17}$	$1.91\times 10^{-64}$	$1.58\times 10^{-68}$
F2	Aver.	$4.36\times 10^{-02}$	$6.00\times 10^{-01}$	$3.84\times 10^{-05}$	$1.76\times 10^{-15}$	$5.24\times 10^{-06}$	$2.26\times 10^{-13}$	$1.57\times 10^{-32}$	$4.18\times 10^{-33}$
	Stdev.	$4.80\times 10^{-02}$	$6.86\times 10^{-02}$	$1.22\times 10^{-04}$	$2.33\times 10^{-15}$	$6.71\times 10^{-07}$	$9.69\times 10^{-15}$	$1.55\times 10^{-33}$	$1.14\times 10^{-33}$
F3	Aver.	$8.59\times 10^{+01}$	$5.19\times 10^{+02}$	$2.39\times 10^{-02}$	$3.48\times 10^{+00}$	$7.03\times 10^{-10}$	$3.27\times 10^{-16}$	$5.02\times 10^{-18}$	$1.08\times 10^{+02}$
	Stdev.	$4.44\times 10^{+01}$	$1.24\times 10^{+02}$	$7.74\times 10^{-02}$	$2.56\times 10^{+00}$	$2.17\times 10^{-10}$	$1.19\times 10^{-17}$	$6.98\times 10^{-18}$	$9.61\times 10^{+01}$
F4	Aver.	$8.45\times 10^{-01}$	$1.07\times 10^{+00}$	$1.96\times 10^{-01}$	$2.63\times 10^{-01}$	$1.07\times 10^{-05}$	$2.51\times 10^{-13}$	$3.51\times 10^{-14}$	$4.53\times 10^{-02}$
	Stdev.	$1.01\times 10^{+00}$	$8.09\times 10^{-02}$	$6.15\times 10^{-01}$	$1.12\times 10^{-01}$	$1.86\times 10^{-06}$	$1.34\times 10^{-14}$	$1.31\times 10^{-13}$	$1.77\times 10^{-01}$
F5	Aver.	$3.85\times 10^{+01}$	$3.89\times 10^{+01}$	$7.45\times 10^{-01}$	$3.81\times 10^{+01}$	$3.37\times 10^{+01}$	$2.89\times 10^{+01}$	$6.02\times 10^{+00}$	$2.73\times 10^{+01}$
	Stdev.	$1.27\times 10^{+01}$	$1.25\times 10^{+00}$	$6.90\times 10^{+00}$	$2.69\times 10^{+01}$	$6.96\times 10^{+01}$	$3.01\times 10^{-02}$	$2.40\times 10^{+00}$	$2.22\times 10^{-01}$
F6	Aver.	$7.08\times 10^{-04}$	$5.69\times 10^{-01}$	$1.14\times 10^{-09}$	$2.45\times 10^{-23}$	$4.48\times 10^{-10}$	$4.93\times 10^{+00}$	**0**	**0**
	Stdev.	$3.05\times 10^{-03}$	$9.65\times 10^{-02}$	$3.65\times 10^{-09}$	$6.62\times 10^{-23}$	$1.60\times 10^{-10}$	$6.58\times 10^{-01}$	**0**	**0**
F7	Aver.	$4.61\times 10^{-02}$	$1.20\times 10^{-03}$	$4.26\times 10^{-02}$	$3.20\times 10^{-03}$	$1.32\times 10^{-03}$	$2.74\times 10^{-04}$	$5.55\times 10^{-04}$	$3.09\times 10^{-03}$
	Stdev.	$1.88\times 10^{-02}$	$2.94\times 10^{-04}$	$1.32\times 10^{-01}$	$1.19\times 10^{-03}$	$9.73\times 10^{-04}$	$9.29\times 10^{-05}$	$1.65\times 10^{-04}$	$7.28\times 10^{-04}$
F8	Aver.	$-4.08\times 10^{+03}$	$-4.39\times 10^{+03}$	$-1.07\times 10^{+03}$	$-6.76\times 10^{+03}$	$-2.96\times 10^{+03}$	$-4.39\times 10^{+03}$	$-1.04\times 10^{+04}$	$-5.21\times 10^{+03}$
	Stdev.	$2.53\times 10^{+02}$	$3.47\times 10^{+02}$	$3.25\times 10^{+03}$	$7.70\times 10^{+02}$	$2.25\times 10^{+02}$	$3.04\times 10^{+02}$	$5.77\times 10^{+02}$	$2.06\times 10^{+03}$
F9	Aver.	$1.49\times 10^{+02}$	$1.57\times 10^{+02}$	$1.99\times 10^{-01}$	$3.28\times 10^{+01}$	$1.30\times 10^{+01}$	$5.69\times 10^{-15}$	$2.47\times 10^{+01}$	$5.44\times 10^{+00}$
	Stdev.	$1.17\times 10^{+01}$	$8.45\times 10^{+00}$	$9.38\times 10^{-01}$	$1.09\times 10^{+01}$	$5.82\times 10^{+00}$	$1.80\times 10^{-14}$	$5.94\times 10^{+00}$	$2.27\times 10^{+00}$
F10	Aver.	$4.44\times 10^{-15}$	$3.64\times 10^{-01}$	$7.97\times 10^{-06}$	$6.98\times 10^{-13}$	$2.20\times 10^{+00}$	$1.92\times 10^{-13}$	$6.93\times 10^{-15}$	$4.44\times 10^{-15}$
	Stdev.	**0**	$2.80\times 10^{-02}$	$2.43\times 10^{-05}$	$1.14\times 10^{-12}$	$7.19\times 10^{-01}$	$4.17\times 10^{-14}$	$1.66\times 10^{-15}$	**0**
F11	Aver.	$2.84\times 10^{-03}$	$7.13\times 10^{-01}$	$9.86\times 10^{-05}$	$1.12\times 10^{-02}$	$3.04\times 10^{-01}$	**0**	**0**	**0**
	Stdev.	$1.32\times 10^{-03}$	$4.16\times 10^{-02}$	$9.86\times 10^{-04}$	$1.25\times 10^{-02}$	$1.58\times 10^{-01}$	**0**	**0**	**0**
F12	Aver.	$5.50\times 10^{-05}$	$5.51\times 10^{-03}$	$4.15\times 10^{-03}$	$2.03\times 10^{-26}$	$1.04\times 10^{-01}$	$3.51\times 10^{-01}$	$1.57\times 10^{-32}$	$1.57\times 10^{-32}$
	Stdev.	$7.78\times 10^{-05}$	$8.54\times 10^{-04}$	$2.52\times 10^{-02}$	$5.27\times 10^{-26}$	$3.20\times 10^{-01}$	$9.59\times 10^{-02}$	$3.16\times 10^{-02}$	$5.57\times 10^{-48}$
F13	Aver.	$5.46\times 10^{-03}$	$6.17\times 10^{-02}$	$4.39\times 10^{-04}$	$1.10\times 10^{-03}$	$7.32\times 10^{-04}$	$1.98\times 10^{+00}$	$1.35\times 10^{-32}$	$1.35\times 10^{-32}$
	Stdev.	$7.45\times 10^{-03}$	$9.98\times 10^{-03}$	$2.16\times 10^{-03}$	$3.35\times 10^{-03}$	$2.79\times 10^{-03}$	$3.36\times 10^{-01}$	$5.57\times 10^{-48}$	$5.57\times 10^{-48}$
F14	Aver.	1.0037	1.0970	0.3948	1.3280	0.9980	0.9983	0.9980	1.2470
	Stdev.	$3.12\times 10^{-02}$	$5.42\times 10^{-01}$	$1.56\times 10^{+00}$	$9.47\times 10^{-01}$	$1.51\times 10^{-16}$	$1.34\times 10^{-03}$	**0**	$9.44\times 10^{-01}$
F15	Aver.	$6.89\times 10^{-04}$	$3.07\times 10^{-04}$	$3.83\times 10^{-04}$	$3.69\times 10^{-04}$	$6.94\times 10^{-04}$	$3.18\times 10^{-04}$	$3.07\times 10^{-04}$	$7.31\times 10^{-04}$
	Stdev.	$1.73\times 10^{-04}$	$3.30\times 10^{-10}$	$2.05\times 10^{-03}$	$2.32\times 10^{-04}$	$3.64\times 10^{-04}$	$8.14\times 10^{-06}$	$7.67\times 10^{-20}$	$2.37\times 10^{-05}$
F16	Aver.	−1.0316	−1.0316	−0.1032	−1.0316	−1.0316	−1.0316	−1.0316	−1.0316
	Stdev.	$6.78\times 10^{-16}$	$6.20\times 10^{-07}$	$3.11\times 10^{-01}$	$6.78\times 10^{-16}$	$1.04\times 10^{-15}$	$5.73\times 10^{-06}$	$6.78\times 10^{-16}$	$6.78\times 10^{-16}$
F17	Aver.	0.3979	0.3979	0.3979	0.4665	0.3979	0.3979	0.3979	0.3979
	Stdev.	**0**	$9.80\times 10^{-06}$	$1.20\times 10^{-01}$	$1.27\times 10^{-01}$	$2.63\times 10^{-15}$	$9.97\times 10^{-05}$	**0**	**0**
F18	Aver.	3.9000	6.4068	3.0000	3.0000	3.0000	3.0020	3.0000	3.9000
	Stdev.	$4.93\times 10^{+00}$	$1.09\times 10^{+01}$	$1.245\times 10^{-10}$	$1.31\times 10^{-15}$	$3.80\times 10^{-14}$	$1.41\times 10^{-03}$	$1.76\times 10^{-15}$	$4.93\times 10^{+00}$
F19	Aver.	−3.8628	−3.8593	−0.3863	−3.7727	−3.8628	−3.8619	−3.8628	−3.8064
	Stdev.	$2.71\times 10^{-15}$	$1.20\times 10^{-02}$	$1.16\times 10^{+00}$	$6.63\times 10^{-02}$	$2.84\times 10^{-15}$	$1.17\times 10{}^{-}03$	$2.71\times 10^{-15}$	$1.96\times 10^{-01}$
F20	Aver.	−3.2784	−3.2863	−0.3251	−2.3324	−3.2190	−3.1088	−3.27	−3.2900
	Stdev.	$5.83\times 10^{-02}$	$5.54\times 10^{-02}$	$9.80\times 10^{-01}$	$3.16\times 10^{-01}$	$4.11\times 10^{-02}$	$7.21\times 10^{-02}$	$5.92\times 10^{-02}$	$5.33\times 10^{-02}$
F21	Aver.	−10.1532	−6.1882	−0.638	−2.3449	−9.0573	−9.1254	−10.1532	−6.7096
	Stdev.	$6.63\times 10^{-15}$	$3.77\times 10^{+00}$	$2.18\times 10^{+00}$	$9.81\times 10^{-01}$	$2.27\times 10^{+00}$	$9.23\times 10^{-01}$	$1.90\times 10^{+00}$	$3.75\times 10^{+00}$
F22	Aver.	−9.5164	−10.1479	−0.7815	−2.2815	−9.8742	−9.7991	−10.4029	−10.4029
	Stdev.	$2.58\times 10^{+00}$	$1.40\times 10^{+00}$	$2.57\times 10^{+00}$	$9.73\times 10^{-01}$	$1.61\times 10^{+00}$	$5.03\times 10^{-01}$	$1.75\times 10^{-15}$	$1.65\times 10^{-15}$
F23	Aver.	−10.3130	−10.5364	−0.8559	−2.3258	−9.919	−10.0764	−10.5364	−10.5364
	Stdev.	$2.88\times 10^{+00}$	$2.22\times 10^{-09}$	$2.76\times 10^{+00}$	$9.13\times 10^{-01}$	$1.91\times 10^{+00}$	$2.96\times 10^{-01}$	$1.62\times 10^{-15}$	$1.81\times 10^{-15}$

**Table 3:** Comparison of the optimization results obtained in Test 2 (d=50).

F	Meas.	GA	PSO	SSA	BOA	HFA	CEFA
F1	Aver.	$4.92\times 10^{-09}$	$5.32\times 10^{-19}$	$4.68\times 10^{-09}$	$2.28\times 10^{-18}$	$3.24\times 10^{-106}$	$2.05\times 10^{-65}$
	Stdev.	$1.91\times 10^{-08}$	$8.24\times 10^{-19}$	$7.90\times 10^{-10}$	$8.51\times 10^{-20}$	$1.64\times 10^{-59}$	$7.97\times 10^{-66}$
F2	Aver.	$1.16\times 10^{-02}$	$1.96\times 10^{-12}$	$4.58\times 10^{-06}$	$3.47\times 10^{+20}$	$4.08\times 10^{-54}$	$1.25\times 10^{-31}$
	Stdev.	$5.01\times 10^{-02}$	$4.69\times 10^{-12}$	$9.23\times 10^{-07}$	$1.90\times 10^{+21}$	$3.36\times 10^{-55}$	$2.66\times 10^{-32}$
F3	Aver.	$2.28\times 10^{-01}$	$1.58\times 10^{+02}$	$5.06\times 10^{-10}$	$2.33\times 10^{-18}$	$3.38\times 10^{-09}$	$5.61\times 10^{+02}$
	Stdev.	$7.19\times 10^{-01}$	$5.26\times 10^{+01}$	$1.55\times 10^{-10}$	$7.58\times 10^{-20}$	$2.92\times 10^{-09}$	$2.98\times 10^{+02}$
F4	Aver.	$2.34\times 10^{-01}$	$2.48\times 10^{+00}$	$1.03\times 10^{-05}$	$1.98\times 10^{-15}$	$1.43\times 10^{-02}$	$1.91\times 10^{+00}$
	Stdev.	$7.34\times 10^{-01}$	$4.73\times 10^{-01}$	$1.58\times 10^{-06}$	$5.10\times 10^{-17}$	$1.86\times 10^{-02}$	$1.89\times 10^{+00}$
F5	Aver.	$2.07\times 10^{+00}$	$7.89\times 10^{+01}$	$6.51\times 10^{+01}$	$4.89\times 10^{+01}$	$2.55\times 10^{+01}$	$3.92\times 10^{+01}$
	Stdev.	$1.14\times 10^{+01}$	$3.40\times 10^{+01}$	$6.03\times 10^{+01}$	$3.00\times 10^{-02}$	$2.27\times 10^{+01}$	$5.17\times 10^{+00}$
F6	Aver.	$1.28\times 10^{-08}$	$5.97\times 10^{-19}$	$3.36\times 10^{-10}$	$9.52\times 10^{+00}$	$2.47\times 10^{-33}$	**0**
	Stdev.	$5.90\times 10^{-08}$	$1.20\times 10^{-18}$	$1.01\times 10^{-10}$	$7.24\times 10^{-01}$	$5.63\times 10^{-33}$	**0**
F7	Aver.	$1.39\times 10^{-01}$	$8.66\times 10^{-03}$	$9.23\times 10^{-04}$	$1.79\times 10^{-04}$	$1.59\times 10^{-03}$	$3.76\times 10^{-03}$
	Stdev.	$4.26\times 10^{-01}$	$2.33\times 10^{-03}$	$8.29\times 10^{-04}$	$6.52\times 10^{-05}$	$4.09\times 10^{-04}$	$9.56\times 10^{-04}$
F8	Aver.	$-1.67\times 10^{+03}$	$-1.13\times 10^{+04}$	$-3.01\times 10^{+03}$	$-5.98\times 10^{+03}$	$-1.61\times 10^{+04}$	$-7.42\times 10^{+03}$
	Stdev.	$5.05\times 10^{+03}$	$1.22\times 10^{+03}$	$2.30\times 10^{+02}$	$4.52\times 10^{+02}$	$7.73\times 10^{+02}$	$4.08\times 10^{+03}$
F9	Aver.	$1.99\times 10^{-01}$	$5.91\times 10^{+01}$	$1.23\times 10^{+01}$	**0**	$6.83\times 10^{+01}$	$1.34\times 10^{+01}$
	Stdev.	$7.75\times 10^{-01}$	$1.34\times 10^{+01}$	$4.20\times 10^{+00}$	**0**	$1.55\times 10^{+01}$	$3.20\times 10^{+00}$
F10	Aver.	$1.76\times 10^{-02}$	$1.20\times 10^{-10}$	$2.26\times 10^{-01}$	$4.20\times 10^{-15}$	$8.70\times 10^{-15}$	$1.98\times 10^{-15}$
	Stdev.	$1.24\times 10^{-01}$	$1.54\times 10^{-10}$	$6.37\times 10^{-01}$	$9.01\times 10^{-16}$	$2.17\times 10^{-15}$	$1.79\times 10^{-16}$
F11	Aver.	$1.48\times 10^{-04}$	$7.55\times 10^{-03}$	$2.80\times 10^{-01}$	**0**	$1.15\times 10^{-03}$	**0**
	Stdev.	$1.48\times 10^{-03}$	$8.76\times 10^{-03}$	$1.20\times 10^{-01}$	**0**	$3.09\times 10^{-02}$	**0**
F12	Aver.	$3.73\times 10^{-03}$	$8.29\times 10^{-03}$	$2.07\times 10^{-02}$	$6.73\times 10^{-01}$	$2.08\times 10^{-02}$	$9.42\times 10^{-33}$
	Stdev.	$1.48\times 10^{-02}$	$2.70\times 10^{-02}$	$7.89\times 10^{-02}$	$1.04\times 10^{-01}$	$1.03\times 10^{-01}$	$2.78\times 10^{-48}$
F13	Aver.	$7.69\times 10^{-04}$	$3.30\times 10^{-03}$	$1.65\times 10^{-11}$	$4.06\times 10^{+00}$	$2.56\times 10^{-03}$	$1.35\times 10^{-32}$
	Stdev.	$4.75\times 10^{-03}$	$5.12\times 10^{-03}$	$5.72\times 10^{-12}$	$7.17\times 10^{-01}$	$4.73\times 10^{-03}$	$5.57\times 10^{-48}$

**Table 4:** Comparison of the optimization results obtained in Test 3 (d=100).

F	Meas.	GA	PSO	SSA	BOA	HFA	CEFA
F1	Aver.	$1.02\times 10^{-02}$	$1.40\times 10^{-05}$	$4.65\times 10^{-09}$	$2.34\times 10^{-18}$	$6.00\times 10^{-44}$	$1.93\times 10^{-44}$
	Stdev.	$3.45\times 10^{-02}$	$1.18\times 10^{-05}$	$8.94\times 10^{-10}$	$6.49\times 10^{-20}$	$9.67\times 10^{-44}$	$6.54\times 10^{-45}$
F2	Aver.	$6.88\times 10^{-01}$	$7.58\times 10^{-04}$	$4.69\times 10^{-06}$	$3.76\times 10^{+46}$	$1.81\times 10^{-29}$	$1.70\times 10^{-21}$
	Stdev.	$2.22\times 10^{+00}$	$2.11\times 10^{-03}$	$1.02\times 10^{-06}$	$8.32\times 10^{+46}$	$1.52\times 10^{-29}$	$2.88\times 10^{-22}$
F3	Aver.	$2.95\times 10^{+00}$	$7.67\times 10^{+03}$	$4.73\times 10^{-10}$	$2.39\times 10^{-18}$	$3.03\times 10^{+03}$	$7.50\times 10^{+03}$
	Stdrv.	$9.16\times 10^{+00}$	$1.70\times 10^{+03}$	$1.95\times 10^{-10}$	$6.96\times 10^{-20}$	$4.07\times 10^{+03}$	$1.84\times 10^{+03}$
F4	Aver.	$2.53\times 10^{-01}$	$8.39\times 10^{+00}$	$1.01\times 10^{-05}$	$2.00\times 10^{-15}$	$5.89\times 10^{+01}$	$1.51\times 10^{+01}$
	Stdev.	$7.75\times 10^{-01}$	$7.99\times 10^{-01}$	$1.51\times 10^{-06}$	$6.00\times 10^{-17}$	$5.13\times 10^{+00}$	$4.30\times 10^{+00}$
F5	Aver.	$1.65\times 10^{+01}$	$2.38\times 10^{+02}$	$1.70\times 10^{+02}$	$9.89\times 10^{+01}$	$1.34\times 10^{+02}$	$1.04\times 10^{+02}$
	Stdev.	$5.37\times 10^{+01}$	$9.49\times 10^{+01}$	$7.30\times 10^{+01}$	$2.74\times 10^{-02}$	$5.26\times 10^{+01}$	$2.47\times 10^{+01}$
F6	Aver.	$3.08\times 10^{-02}$	$8.74\times 10^{-06}$	$3.57\times 10^{-10}$	$2.23\times 10^{+01}$	$2.17\times 10^{-31}$	**0**
	Stdev.	$1.06\times 10^{-01}$	$8.32\times 10^{-06}$	$1.39\times 10^{-10}$	$9.59\times 10^{-01}$	$2.40\times 10^{-31}$	**0**
F7	Aver.	$3.57\times 10^{-01}$	$6.37\times 10^{-02}$	$8.10\times 10^{-04}$	$1.79\times 10^{-04}$	$1.26\times 10^{-02}$	$9.36\times 10^{-03}$
	Stdev.	$1.11\times 10^{+00}$	$1.09\times 10^{-02}$	$5.87\times 10^{-04}$	$6.49\times 10^{-05}$	$2.91\times 10^{-03}$	$1.46\times 10^{-03}$
F8	Aver.	$-2.81\times 10^{+03}$	$-2.10\times 10^{+04}$	-$3.06\times 10^{+03}$	$-8.52\times 10^{+03}$	$-3.00\times 10^{+04}$	$-9.24\times 10^{+03}$
	Stdev.	$8.47\times 10^{+03}$	$2.19\times 10^{+03}$	$3.55\times 10^{+02}$	$7.06\times 10^{+02}$	$1.32\times 10^{+03}$	$3.90\times 10^{+02}$
F9	Aver.	$2.59\times 10^{+00}$	$1.29\times 10^{+02}$	$4.71\times 10^{+01}$	**0**	$2.28\times 10^{+02}$	$3.91\times 10^{+01}$
	Stdev.	$8.08\times 10^{+00}$	$2.03\times 10^{+01}$	$1.43\times 10^{+01}$	**0**	$4.64\times 10^{+01}$	$5.09\times 10^{+00}$
F10	Aver.	$1.12\times 10^{-01}$	$1.54\times 10^{-02}$	$2.66\times 10^{-01}$	$4.44\times 10^{-15}$	$2.43\times 10^{-01}$	$4.44\times 10^{-15}$
	Stdev.	$3.44\times 10^{-01}$	$6.60\times 10^{-02}$	$6.26\times 10^{-01}$	$5.32\times 10^{-16}$	$5.12\times 10^{-01}$	$4.01\times 10^{-16}$
F11	Aver.	$2.30\times 10^{-04}$	$7.21\times 10^{-03}$	$3.02\times 10^{-01}$	**0**	$3.37\times 10^{-03}$	**0**
	Stdev.	$7.63\times 10^{-04}$	$1.39\times 10^{-02}$	$1.03\times 10^{-01}$	**0**	$5.62\times 10^{-03}$	**0**
F12	Aver.	$2.18\times 10^{-03}$	$1.66\times 10^{-02}$	$6.22\times 10^{-02}$	$9.51\times 10^{-01}$	$7.61\times 10^{-02}$	$2.92\times 10^{-04}$
	Stdev.	$8.86\times 10^{-03}$	$3.03\times 10^{-02}$	$1.51\times 10^{-01}$	$7.96\times 10^{-02}$	$1.16\times 10^{-01}$	$1.60\times 10^{-03}$
F13	Aver.	$2.71\times 10^{-03}$	$7.72\times 10^{-03}$	$7.32\times 10^{-04}$	$9.98\times 10^{+00}$	$9.08\times 10^{-02}$	$1.35\times 10^{-32}$
	Stdev.	$9.66\times 10^{-03}$	$1.04\times 10^{-02}$	$2.79\times 10^{-03}$	$6.30\times 10^{-03}$	$3.28\times 10^{-01}$	$5.57\times 10^{-48}$

**Table 5:** Experimental results of different numbers of iterations for FA and CE operators in CEFA.

$N_{1}$	$N_{2}$	30	50	100	200	500	1000
1	$F1_{\min}$	$6.59\times 10^{+00}$	$8.94\times 10^{-02}$	$3.32\times 10^{-04}$	$6.03\times 10^{-07}$	$4.95\times 10^{-15}$	$3.62\times 10^{-28}$
	*T*	0.01	0.02	0.05	0.10	0.24	0.48
5	$F1_{\min}$	$8.49\times 10^{-11}$	$6.98\times 10^{-21}$	$3.08\times 10^{-45}$	$4.43\times 10^{-95}$	0	0
	*T*	0.03	0.04	0.08	0.15	0.34	0.76
10	$F1_{\min}$	$7.16\times 10^{-20}$	$2.80\times 10^{-36}$	$4.30\times 10^{-76}$	0	0	0
	*T*	0.04	0.05	0.11	0.31	0.76	2.10
30	$F1_{\min}$	$8.83\times 10^{-40}$	$6.80\times 10^{-69}$	0	0	0	0
	*T*	0.14	0.21	0.42	0.79	2.16	4.70
50	$F1_{\min}$	$6.84\times 10^{-51}$	$1.32\times 10^{-87}$	0	0	0	0
	*T*	0.24	0.31	0.57	1.15	3.43	6.35
100	$F1_{\min}$	$8.11\times 10^{-68}$	0	0	0	0	0
	*T*	0.35	0.64	1.19	2.19	6.57	12.03
200	$F1_{\min}$	$3.10\times 10^{-86}$	0	0	0	0	0
	*T*	0.54	1.07	2.10	4.61	12.75	24.10
300	$F1_{\min}$	$8.97\times 10^{-98}$	0	0	0	0	0
	*T*	1.13	1.48	3.01	6.80	18.56	38.75

## Appendix A

See Table A1.

**Table A1:** The definition of benchmark functions.

Function	Dim	Range	$F_{\min}$	Type
$F1\left(x\right)=\sum_{i=1}^{n}x_{i}^{2}$	30,50,100	[−100,100]	0	Unimodal
$F2\left(x\right)=\sum_{i=1}^{n}\left|x_{i}\right|+\prod_{i=1}^{n}\left|x_{i}\right|$	30,50,100	[−10,10]	0	Unimodal
$F3\left(x\right)=\sum_{i=1}^{n}{\left(\sum_{j=1}^{i}x_{j}\right)}^{2}$	30,50,100	[−100,100]	0	Unimodal
$F4\left(x\right)=\max_{i}\left\{\left|x_{i}\right|,1\leq i\leq n\right\}$	30,50,100	[−100,100]	0	Unimodal
$F5\left(x\right)=\sum_{i=1}^{n-1}\left[100{\left(x_{i+1}-x_{i}^{2}\right)}^{2}+{\left(x_{i}-1\right)}^{2}\right]$	30,50,100	[−30,30]	0	Unimodal
$F6\left(x\right)=\sum_{i=1}^{n}{\left(\left[x_{i}+0.5\right]\right)}^{2}$	30,50,100	[−100,100]	0	Unimodal
$F7\left(x\right)=\sum_{i=1}^{n}ix_{i}^{4}+random\left[0,1\right)$	30,50,100	[−1.28,1.28]	0	Unimodal
$F8\left(x\right)=\sum_{i=1}^{n}-x_{i}\sin\left(\sqrt{\left|x_{i}\right|}\right)$	30,50,100	[−500,500]	−418.9829×n	Multimodal
$F9\left(x\right)=\sum_{i=1}^{n-1}\left[x_{i}^{2}-10\cos\left(2\pi x_{i}\right)+10\right]$	30,50,100	[−5.12,5.12]	0	Multimodal
$F10\left(x\right)=-20\exp\left(-0.2\sqrt{\frac{1}{n}\sum_{i=1}^{n}x_{i}^{2}}\right)-\exp\left(\frac{1}{n}\sum_{i=1}^{n}\cos\left(2\pi x_{i}\right)\right)+20+e$	30,50,100	[−32,32]	0	Multimodal
$F11\left(x\right)=\frac{1}{4000}\sum_{i=1}^{n}x_{i}^{2}-\prod_{i=1}^{n}\cos\left(\frac{x_{i}}{\sqrt{i}}\right)+1$	30,50,100	[−600,600]	0	Multimodal
$F12\left(x\right)=\frac{\pi }{n}\left\{10\sin^{2}\left(\pi y_{1}\right)+\sum_{i=1}^{n-1}{\left(y_{i}-1\right)}^{2}\left[1+10\sin^{2}\left(\pi y_{i+1}\right)+{\left(y_{n}+1\right)}^{2}\right]\right\}+\sum_{i=1}^{n}u\left(x_{i},10,100,4\right)$	30,50,100	[−50,50]	0	Multimodal
$y_{i}=1+\frac{x_{i}+1}{4}$				
$u\left(x_{i},a,k,m\right)=\left\{\begin{array}{cc} k{\left(x_{i}-a\right)}^{m}, & x_{i}>a\\ 0, & -a\leq x_{i}\leq a\\ k{\left(-x_{i}-a\right)}^{m}, & x_{i}<-a \end{array}\right.$				
$F13\left(x\right)=0.1\left\{\sin^{2}\left(3\pi x_{1}\right)+\sum_{i=1}^{n}{\left(x_{i}-1\right)}^{2}\left[1+\sin^{2}\left(3\pi x_{i}+1\right)\right]\right\}+\sum_{i=1}^{n}u\left(x_{i},5,100,4\right)$	30,50,100	[−50,50]	0	Multimodal
$F14\left(x\right)={\left(\frac{1}{500}+\sum_{j=1}^{25}\frac{1}{j+\sum_{i=1}^{2}{\left(x_{i}-a_{ij}\right)}^{6}}\right)}^{-1}$	2	[−65.536,65.536]	1	Multimodal
$F15\left(x\right)=\sum_{i=1}^{11}{\left[a_{i}-\frac{x_{1}\left(b_{i}^{2}+b_{i}x_{2}\right)}{b_{i}^{2}+b_{i}x_{3}+x_{4}}\right]}^{2}$	4	[−5,5]	0.00030	Multimodal
$F16\left(x\right)=4x_{1}^{2}-2.1x_{1}^{4}+\frac{1}{3}x_{1}^{6}+x_{1}x_{2}-x2^{2}+4x_{2}^{2}$	2	[−5,5]	−1.0316	Multimodal
$F17\left(x\right)={\left(x_{2}-\frac{5.1}{4\pi }x_{1}^{2}+\frac{5}{\pi }x_{1}-6\right)}^{2}+10\left(1-\frac{1}{8\pi }\right)\cos x_{1}+10$	2	[−5,5]	0.398	Multimodal
$F18\left(x\right)=\left[1+{\left(x_{1}+x_{2}+1\right)}^{2}\left(19-14x_{1}+3x_{1}^{2}-14x_{2}+6x_{1}x_{2}+3x_{2}^{2}\right)\right]\times \left[30+\left(2x_{1}-3x_{2}\right)\left(18-32x_{1}+12x_{1}^{2}+48x_{2}-36x_{1}x_{2}+27x_{2}^{2}\right)\right]$	2	[−5,5]	3	Multimodal
$F19\left(x\right)=-\sum_{i=1}^{4}c_{i}\exp\left(-\sum_{j=1}^{3}a_{ij}{\left(x_{j}-p_{ij}\right)}^{2}\right)$	3	[1,3]	−3.86	Multimodal
$F20\left(x\right)=-\sum_{i=1}^{4}c_{i}\exp\left(-\sum_{j=1}^{6}a_{ij}{\left(x_{j}-p_{ij}\right)}^{2}\right)$	6	[0,1]	−3.32	Multimodal
$F21\left(x\right)=-\sum_{i=1}^{5}{\left[\left(X-a_{i}\right){\left(X-a_{i}\right)}^{T}+c_{i}\right]}^{-1}$	4	[0,10]	−10.1532	Multimodal
$F22\left(x\right)=-\sum_{i=1}^{7}{\left[\left(X-a_{i}\right){\left(X-a_{i}\right)}^{T}+c_{i}\right]}^{-1}$	4	[0,10]	−10.4028	Multimodal
$F23\left(x\right)=-\sum_{i=1}^{10}{\left[\left(X-a_{i}\right){\left(X-a_{i}\right)}^{T}+c_{i}\right]}^{-1}$	4	[0,10]	−10.5363	Multimodal
